# Species coexistence and niche interaction between sympatric giant panda and Chinese red panda: A spatiotemporal approach

**DOI:** 10.1002/ece3.9937

**Published:** 2023-04-21

**Authors:** Bin Feng, Wenke Bai, Xueyang Fan, Mingxia Fu, Xinqiang Song, Jingyi Liu, Weirui Qin, Jindong Zhang, Dunwu Qi, Rong Hou

**Affiliations:** ^1^ Chengdu Research Base of Giant Panda Breeding Sichuan Key Laboratory of Conservation Biology for Endangered Wildlife Chengdu China; ^2^ Key Laboratory of Southwest China Wildlife Resources Conservation China West Normal University Nanchong China; ^3^ Administration of Daxiangling Nature Reserve Yaan China

**Keywords:** *Ailuropoda melanoleuca*, *Ailurus styani*, coexistence mechanism, Daxiangling Mountains, spatiotemporal niche

## Abstract

The giant panda (*Ailuropoda melanoleuca*) and the Chinese red panda (*Ailurus styani*) are distributed in the same region in the mountain forest ecosystem on the eastern edge of the Qinghai Tibet Plateau and share the same food sources. In order to understand how sympatric giant pandas and Chinese red pandas maintain interspecific relationships to achieve stable coexistence, we used species distribution models and diurnal activity rhythms to analyze the spatial and temporal niche characteristics of giant pandas and Chinese red pandas in the Daxiangling Mountain system based on 187 camera traps data. The results show that: (1) In the Daxiangling Mountains, the total area of suitable habitats for giant pandas and Chinese red pandas is 717.61 km^2^ and 730.00 km^2^, respectively, accounting for 17.78% and 18.25%, respectively, of the study area. (2) The top five environmental factors contributing to the model of giant panda and Chinese red panda are precipitation seasonality, temperature seasonality, distance to the road, and elevation and vegetation type. (3) The total overlapping area of suitable habitats for giant pandas and Chinese red pandas is 342.23 km^2^, of which the overlapping area of highly suitable habitats is 98.91 km^2^. The overlapping index of suitable habitats is 0.472, and the overlapping index of highly suitable habitats is 0.348, which indicates that the two achieve spatial niches are separated to achieve stable coexistence. (4) The overlapping index of the daily activity rhythm of giant panda and Chinese red panda is 0.87, which is significantly different (*p* < .05). The existence of Chinese red panda will significantly affect the daily activity rhythm of giant panda (*p* < .001). This research can provide scientific reference for the researches about population and habitat protection of giant pandas and Chinese red pandas, so as to understand the driving mechanism of resource allocation and population dynamics of sympatric species.

## Graphical abstract

In order to understand how sympatric giant pandas and Chinese red pandas maintain interspecific relationships to achieve stable coexistence, we used species distribution models and diurnal activity rhythms to analyze the spatial and temporal niche characteristics of giant pandas and Chinese red pandas in the Daxiangling Mountain system based on 187 infrared camera data. The research results can provide scientific reference for the researches about population and habitat protection of giant pandas and Chinese red pandas, and provide a theoretical basis for the study on the coexistence mechanism of sympatric species, so as to understand the driving mechanism of resource allocation and population dynamics of sympatric species.

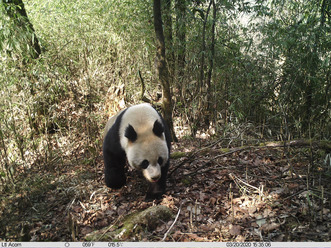

## 1 INTRODUCTION

Revealing the coexistence mechanism of sympatric species with similar niches has always been a research hotspot in community ecology (Davies et al., 2007). The long‐term stable coexistence of sympatric species is based on the niche differentiation between species. The niche is multidimensional, and species can adjust their ecological range in various dimensions through adaptation or behavior change in interspecific competition to maximize benefits and reduce the intensity of competition between species (Bruno et al., 2003). For sympatric closely related species, the differentiation and overlap of niches may more significantly determine whether and in what ways they coexist due to similar physiological needs and behavioral characteristics (Hardin, 1960). Among the many dimensions of niche, space and time are the two most critical dimensions (Cusack et al., 2017). The niches' selection of sympatric species in spatial and temporal dimensions reflects the physiological and ecological needs of species and the interaction between species, which is the basis for exploring the coexistence mechanism of species, and is significantly important for revealing the community structure and protecting regional biodiversity.

**FIGURE 1:**
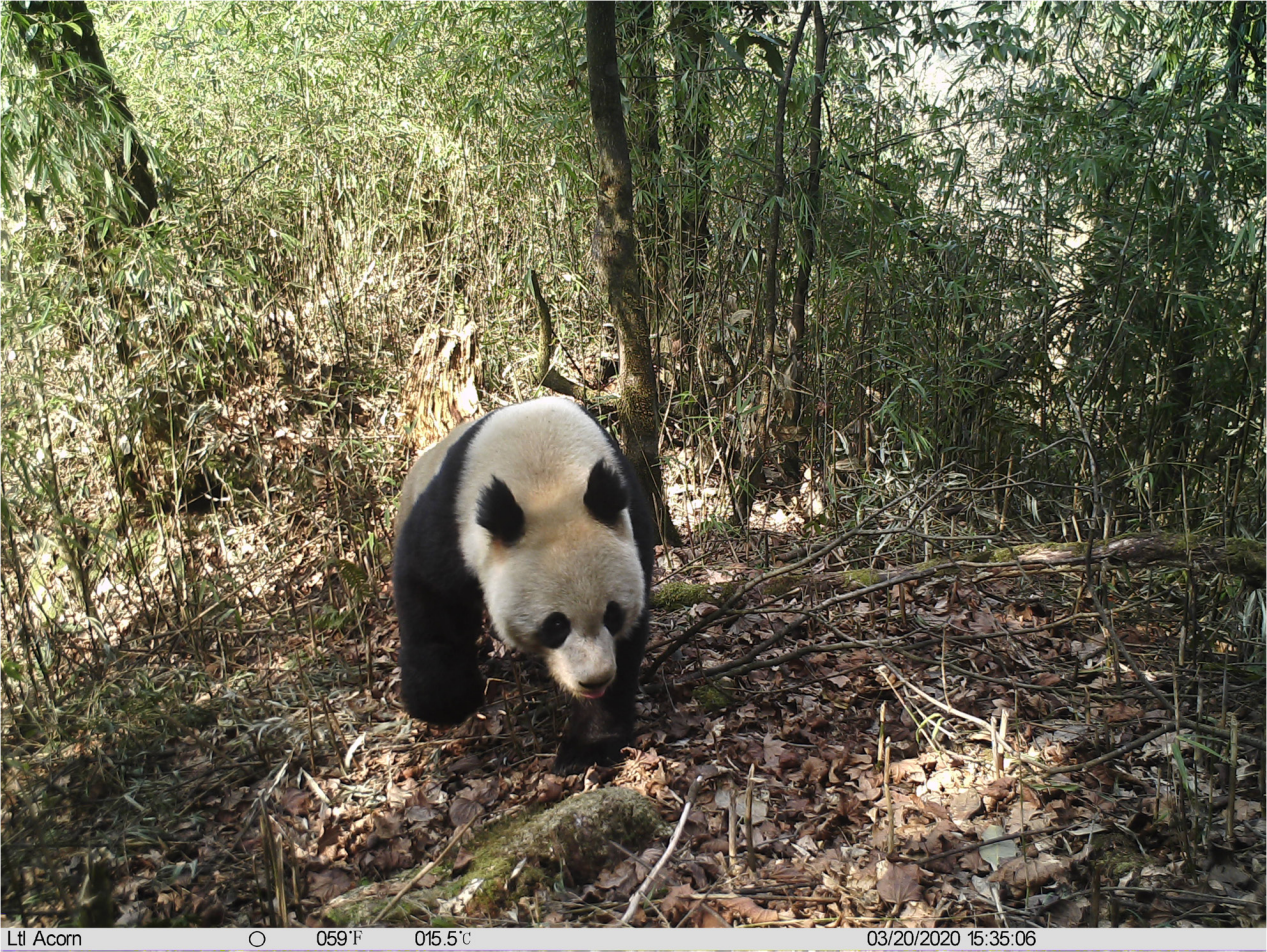
Giant panda (Photographed in Daxiangling Nature Reserve).

**FIGURE 2:**
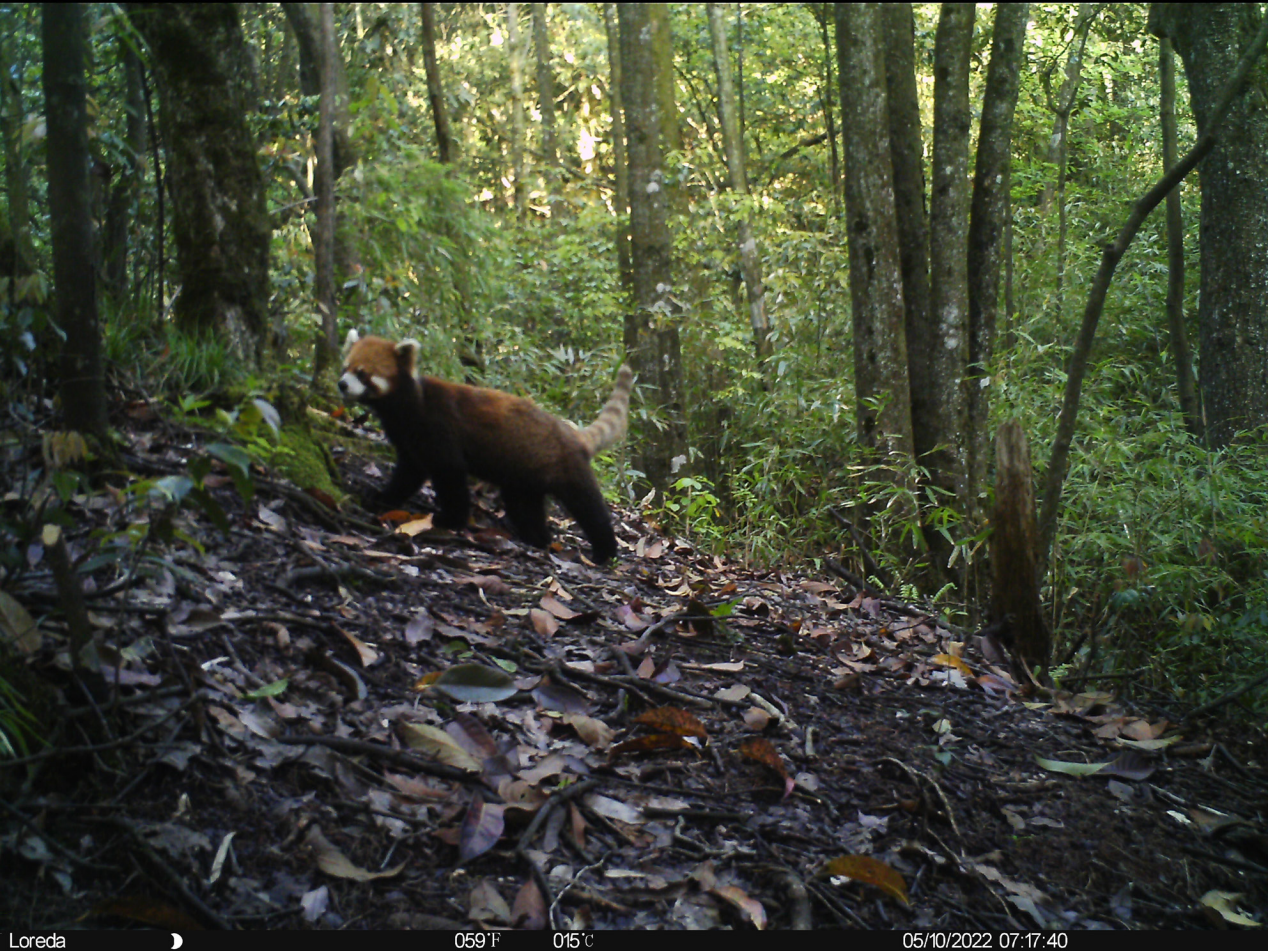
Chinese red panda (Photographed in Daxiangling Nature Reserve).

Giant pandas (*Ailuropoda melanoleuca*; Figure 1) and Chinese red pandas (*Ailurus styani*; Figure 2) belong to Carnivora Ursidae and Ailuridae, respectively (Hu et al., 2020). They play an important ecological role in the distributed habitat, in promoting vegetation community renewal, and in maintaining community structure and species diversity (Ripple et al., 2014). The Chinese red panda is listed as an endangered species by the International Union for the Conservation of Nature (IUCN), even more dangerous than the giant panda. The status of the giant panda has recently changed from “endangered” to “vulnerable” (IUCN, 2020). Similar to the giant panda, the Chinese red panda is also a highly specialized bamboo feeder (Dong et al., 2021; Wei et al., 1999). However, under multiple pressures such as climate change, habitat loss, fragmentation and degradation, the populations, and habitats of both giant panda and Chinese red panda have experienced severe declines, and other associated anthropogenic disturbances (e.g., grazing, resource collection, development activities, road, farming, town expansion.) increasingly aggravate the situation (Cohen et al., 2020; Masel, 2011; Panetta et al., 2018; Wanghe et al., 2020). According to China's fourth giant panda survey, there were 1864 giant pandas in the wild by the end of 2013 (State Forestry Administration, 2015). By the end of the last century, it is estimated that the number of Chinese red pandas may have decreased by 40% due to large‐scale habitat loss, increased human activities, and poaching. At present, the populations of giant pandas and Chinese red pandas are mainly distributed in the mountain forest ecosystem on the eastern edge of the Qinghai Tibet Plateau, especially in Qionglai, Daxiangling, and other mountains. These places have relatively complete protection measures, and the mountain forest ecosystem is well preserved in most areas (Zhuang et al., 2021).

The giant pandas and Chinese red pandas formed an obvious phenomenon of sympatric distribution (Dong et al., 2021; Wang, Yang, et al., 2021). In order to understand how sympatric giant pandas and Chinese red pandas maintain interspecific relationships to achieve stable coexistence, we used species distribution models and diurnal activity rhythms to analyze the spatial and temporal niche characteristics of giant pandas and Chinese red pandas in the Daxiangling Mountain system based on 187 camera traps data. Our research results include the distribution of suitable habitat in the spatial niche, the influence factors of suitable habitat, and the overlap of spatial niche. And the daily activity rhythm, the overlap of activity rhythm, and the interaction relationship in the temporal niche were also investigated. The results of this research can provide scientific reference for the population and habitat protection of giant pandas and Chinese red pandas, and provide a theoretical basis for the study on the coexistence mechanism of sympatric species, so as to understand the driving mechanism of resource allocation and population dynamics of sympatric species.

## 2 MATERIALS AND METHODS

### 2.1 Study area

**FIGURE 3:**
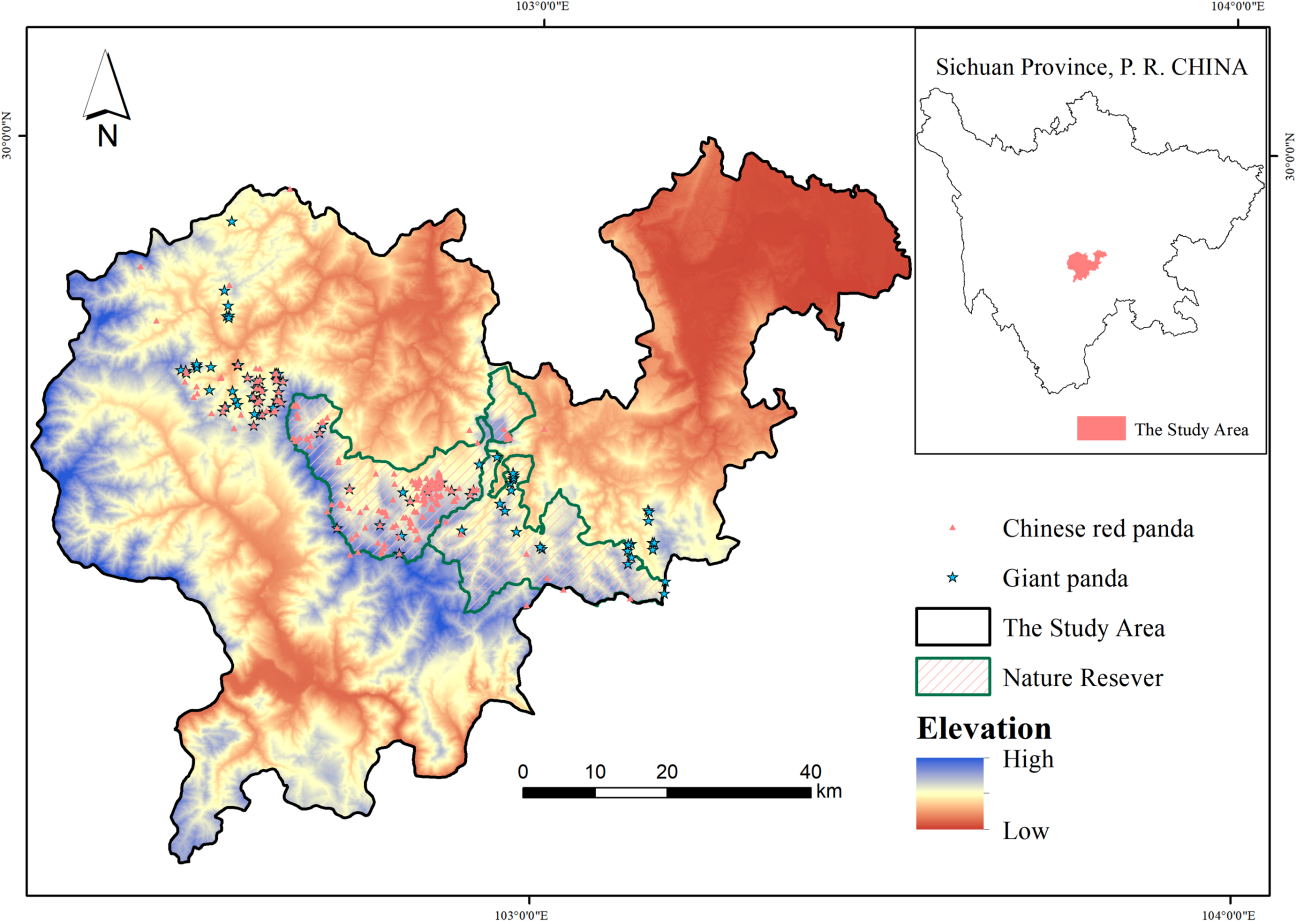
Species occurrence records and study area.

Daxiangling mountain (102° 19~103° 16 E, 29° 24~ 29° 56 N) is located at the eastern edge of the Qinghai Tibet Plateau, covering an area of about 4000 km^2^. The terrain in the mountain system is steep and towering with the highest elevation of more than 3500 m. The water system in Daxiangling Mountains is well developed. The main rivers are Minjiang River and Dadu River. There are many tributaries that provide sufficient water for wildlife. The climate of Daxiangling Mountains is subtropical, with an average annual temperature of about 18°C and an average annual precipitation of about 2000 mm (Zhao et al., 2017). Suitable geographical and climatic conditions have created rich biodiversity. There are many wild animals distributed in the mountains, including giant pandas, Chinese red pandas, Sichuan takins (*Pantholops hodgsoni*), Asian black bears (*Ursus thibetanus*), Temminck's tragopan (*Tragopan tamminckii*), and other rare wild animals (Wang, Yang, et al., 2021). In addition to numerous wild animals, Daxiangling Mountains also have rich and highly diversified vegetation, including *Davidia involucrata* Baill, *Taxus Linn*, *Kingdonia uniflora* Balf, *Tetracentron sinensis* Oliv, Michelia wilsonii Finet et Gagn, Abies fabri (Mast.) Craib and other rare wild plants. It is an ideal habitat for animals and plants (Figure 3).

### 2.2 Spatial niche analysis

We predict the potentially suitable habitats for giant pandas and Chinese red pandas based on the maximum entropy (Maxent) model (Phillips et al., 2006). Maxent is a machine learning method. Because of its high prediction accuracy, it has been widely used in wildlife habitat mapping (Elith et al., 2006; Li et al., 2019). The input data of the model consist of the occurrence records of giant pandas and Chinese red pandas and five types of environmental variables. The prediction effect of the model was evaluated using the ROC curve, namely the area value under the AUC (area under the curve; Huang et al., 2020).

#### 2.2.1 Occurrence points of species

Species occurrence point data used for model construction were extracted from the fourth National Giant Panda Survey and 187 infrared camera data (2014–2020). We eliminated the recurring points in the rectangular area of 1 km^2^ to avoid spatial autocorrelation in the process of model operation (Yang et al., 2017).

#### 2.2.2 Environmental variables and data processing

Five types of environmental variables, including vegetation, water source, disturbance, terrain, and climate, were selected to participate in the model construction. Vegetation data include the normalized difference vegetation index (NDVI) and vegetation types. The NDVI data were extracted from MODIS images derived from the NASA dataset (https://modis.gsfc.nasa.gov). The vegetation type data include 12 kinds of vegetation types extracted from the National Qinghai Tibet Plateau Scientific Data Center (http://data.tpdc.ac.cn). The water source data are the European distance layer of the distribution of rivers and lakes in Daxiangling Mountains calculated in ArcGIS10.4.1, which reflects the distance from each grid to the nearest water source. The Euclidean distance layer of Daxiangling mountain system road calculated in ArcGIS10.4.1 for interference data reflects the distance from each grid to the nearest road. Nineteen bioclimatic factors were selected from the World Climate Database (http://www.worldclim.org). Topographic factors include elevation, slope type, slope position, aspect, and slope, which are extracted from the Digital Terrain Elevation Model (DEM) and derived from the geospatial data cloud platform of Computer Network Information Center of Chinese Academy of Sciences (http://www.gscloud.cn).

Based on the BILINEAR method (continuous variable) and NEAREST method (discrete variable), we uniformly resampled 25 environment variables in ArcGIS10.4.1 to 30 m resolution. To avoid multicollinearity caused by environmental variables, the Pearson correlation coefficient matrix between environmental variables was obtained by Band Collection Statistics tool, and environmental variables with a correlation coefficient >0.7 were removed (Huang et al., 2020). Then, use the remaining variables to build a preanalysis model. According to the analysis results of the model's contribution value, remove the environment variables with contribution <1 (Viña et al., 2010), and finally screen nine environment variables into the MaxEnt model (Table 1).

**TABLE 1:** Environmental variables used in the models and their sources.

Environmental variables	Source	Type of variable
Bio15	http://www.worldclim.org	Continuous variables
Bio7	http://www.worldclim.org	Continuous variables
Bio4	http://www.worldclim.org	Continuous variables
Distance to the river	Calculate Euclidean distances in ArcGIS	Continuous variables
Distance to the road	Calculate Euclidean distances in ArcGIS	Continuous variables
Slope	http://www.gscloud.cn/	Continuous variables
Elevation	http://www.gscloud.cn/	Continuous variables
NDVI	https://modis.gsfc.nasa.gor	Continuous variables
Vegetation type	http://data.tpde.ac.cn	Discrete variables

#### 2.2.3 Mapping suitable habitat of species

The appearance points of giant panda and Chinese red panda, and environmental variables were input into the software MaxEnt3.4.1 to establish the model. The model was set with 75% training set, which was used to build the prediction model, and the remaining 25% was used to verify the prediction results (Wang et al., 2023). The giant panda and the Chinese red panda were bootstraps repeated for five times, respectively, to ensure the accuracy of the model. Other parameters were set by default, and the average value of MaxEnt output results after five times of repetition was taken as the final model result. According to the maximum training sensitivity plus specificity (MaxSS) and the mean value of the habitat suitability index of the suitable habitat, the habitats were divided into nonsuitable habitat, subsuitable habitat, and highly suitable habitat (Wang, Winkler, et al., 2021).

#### 2.2.4 Niche overlap analysis

Based on the prediction results of MaxEnt model, the spatial niche overlap index was used to show the degree of niche overlap between giant pandas and Chinese red pandas. The total habitat overlap area of giant pandas and Chinese red pandas was divided by the square root of the product of their total habitat area to calculate the spatial niche overlap index (Bai et al., 2022, 1). The index is between 0–1. A higher value of this index indicates the higher spatial niche overlap between species. The spatial niche overlap index of giant panda and Chinese red panda in suitable habitat and highly suitable habitat was calculated, respectively, to show the difference of their spatial competition degree in suitable habitat and highly suitable habitat.

(1) $$\;\mathrm{OIa}=\frac{\mathrm{Oij}}{\sqrt{\mathrm{Oi}*\mathrm{Oj}}}$$

### 2.3 Temporal niche analysis

#### 2.3.1 Data processing

The species identification and sorting of wild animals in the infrared camera photos of Daxiangling Mountains were carried out. In order to avoid overestimating the species detection rate, the shooting records of the same species in the same camera site for consecutive 30 min (±1 min) were recorded as an independent effective record (O'Brien, 2008). Finally, a total of 1425 photos/videos of giant panda infrared cameras were used, 245 of which were recorded independently and effectively, and 3361 photos/videos of Chinese giant pandas were recorded independently and effectively, 833 of which were recorded independently and effectively. In order to analyze the response of the activity rhythm of giant pandas and Chinese red pandas to their distribution in the same field, the site data were grouped by whether giant pandas and Chinese red pandas have photographed another species at the same camera site. The occurrence sites of giant pandas were divided into two groups: the presence of Chinese red pandas and the absence of Chinese red pandas. Similarly, the occurrence sites of Chinese red pandas were divided into two groups: the presence of giant pandas and the absence of giant pandas.

#### 2.3.2 Analysis of daily activity rhythm

Random samples were selected from the independent and effective detection photos of giant panda and Chinese red panda. A daily activity rhythm model was established based on kernel density estimation to analyze the characteristics and interaction of the daily activity rhythm of giant panda and Chinese red panda. This method assumes that the probability of target animals being shot by an infrared camera in a specific period of time is proportional to its activity intensity, and each independent and effective shooting is an independent event, which is a random sampling of the probability distribution function of animals being shot by an infrared camera. Therefore, the probability density function of this distribution can be used to reflect the diurnal activity rhythm of animals (Ridout & Linkie, 2009). At the same time, the similarity between the nuclear density curves of giant panda and Chinese red panda was compared to estimate the symmetric overlap degree of their daily activity rhythms, which ranged from 0 (no overlap) to 1 (complete overlap). The R package “overlap” was used to draw the nuclear density curve of species and calculate the overlap index (Meredith & Ridout, 2017). The R package “activity” was used to compare the difference in daily activity rhythm between giant pandas and Chinese red pandas, and the significance level was set as .05 (Rowcliffe, 2014). Statistical analysis and mapping were carried out in R 4.0.3.

## 3 RESULTS

### 3.1 Spatial niche analysis

#### 3.1.1 Prediction of suitable habitat

**FIGURE 4:**
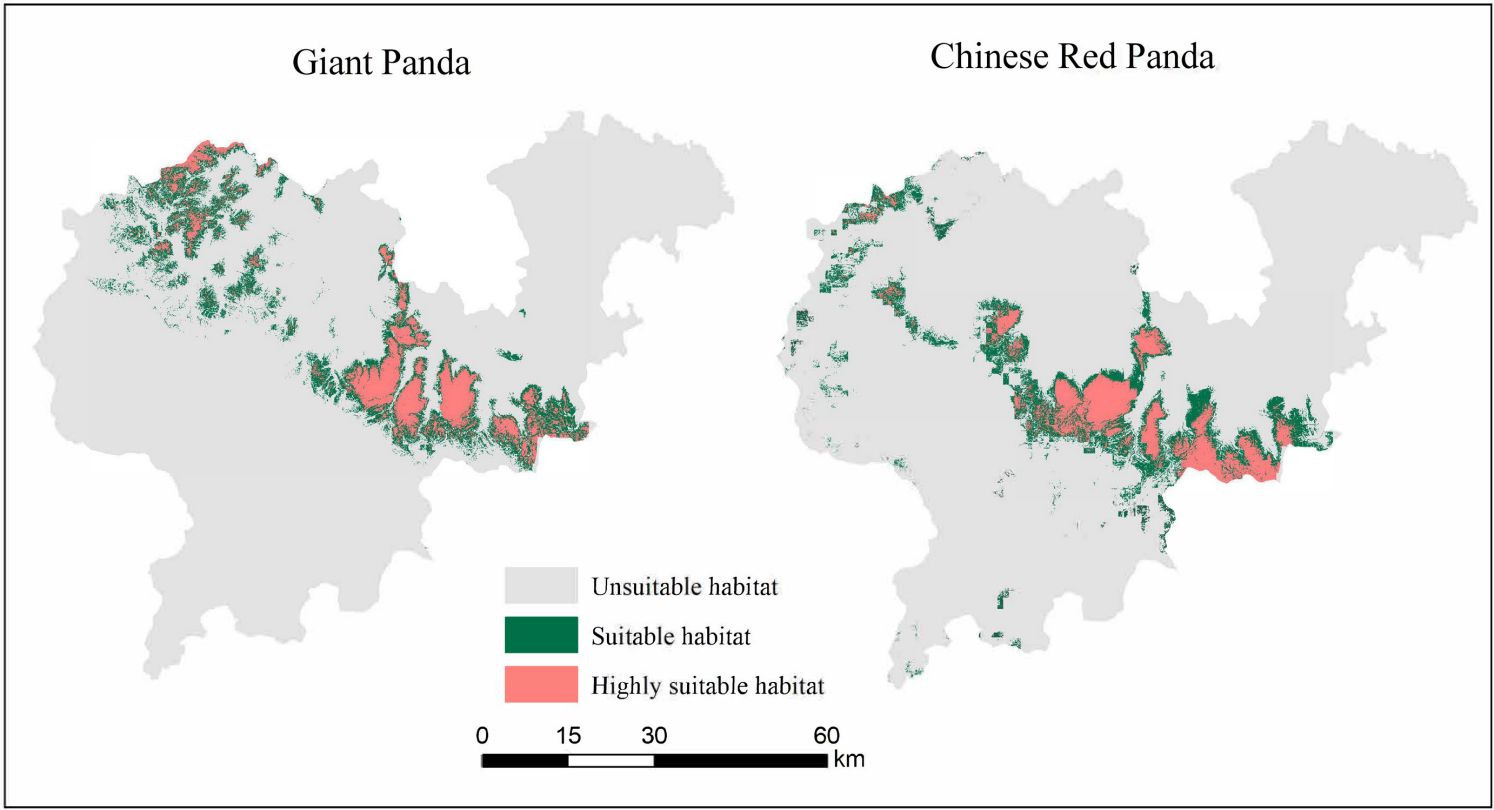
Suitable habitat of giant pandas and Chinese red pandas.

The Maxent model results of giant pandas and Chinese red pandas showed that the AUC values of the MaxEnt model of giant pandas and Chinese red pandas were 0.952 and 0.934, respectively, which indicated that the prediction of the MaxEnt model of giant pandas and Chinese red pandas were accurate and can be used for habitat analysis of giant pandas and Chinese red pandas. The maximum training sensitivity plus specificity (MaxSS) of MaxEnt model results of giant panda and Chinese red panda were 0.289 and 0.237, respectively, and the mean value of the habitat suitability index of the suitable habitat were 0.533 and 0.476, respectively. According to the above threshold, the MaxEnt model prediction results of giant pandas and Chinese red pandas were divided into highly suitable habitat, subsuitable habitat, and unsuitable habitat. The results showed that in the Daxiangling Mountains, the habitat of giant pandas (HSI > 0.289) had a total area of 717.61 km^2^, accounting for 17.78% of the total area of the study area; The area of highly suitable habitat (HSI > 0.533) was 298.64 km^2^, accounting for 7.4% of the study area; The area of subsuitable habitat (0.289 < HSI < 0.533) was 418.97 km^2^, accounting for 10.38% of the total area of the study area. The habitat of the Chinese red panda (HSI > 0.237) had a total area of 730.00 km^2^, accounting for 18.25% of the total area of the study area. Among them, the area of highly suitable habitat (HSI > 0.476) was 296.80 km^2^, accounting for 7.42% of the total area of the study area; The area of subsuitable habitat (0.237 < HSI < 0.476) was 433.20 km^2^, accounting for 10.83% of the total area of the study area. The suitable habitats for giant pandas and Chinese red pandas were mainly distributed in the middle and east of the Daxiangling Mountains. In addition, giant pandas also had a large but severely fragmented suitable habitat in the northwest of the mountain system (Figure 4).

#### 3.1.2 Impact of environmental factors on habitat

**FIGURE 5:**
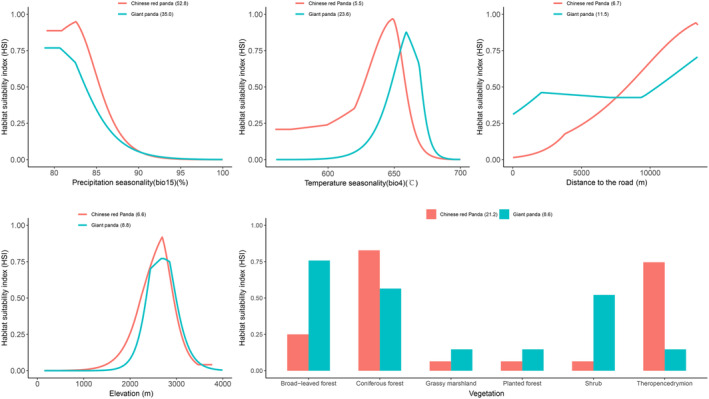
Response curves of habitat suitability of giant pandas and Chinese red pandas on environment variables and contribution value of environment variables.

Among the environmental variables, the top five environmental factors contributing to the giant panda model and the Chinese red panda model were precision seasonality (Bio15), temperature seasonality (Bio4), distance to the road, elevation, and vegetation type, with cumulative contributions of 87.5 and 92.8, respectively. It showed that these environmental factors were important factors for the habitat suitability distribution of giant pandas and Chinese red pandas. The suitable habitats for giant pandas were mainly distributed in coniferous forest, broad‐leaved forest, and shrub habitats with Bio15 < 85 and Bio4 between 645–675 and 2300–3200 m above sea level, which are far from the road. The suitable habitats for Chinese red pandas were mainly distributed in coniferous forests and mixed coniferous and broad‐leaved forests with Bio15 < 87 and Bio4 between 625–660 and 2200–3200 m above sea level, which are far from the road (Figure 5).

#### 3.1.3 Habitat overlaps

**FIGURE 6:**
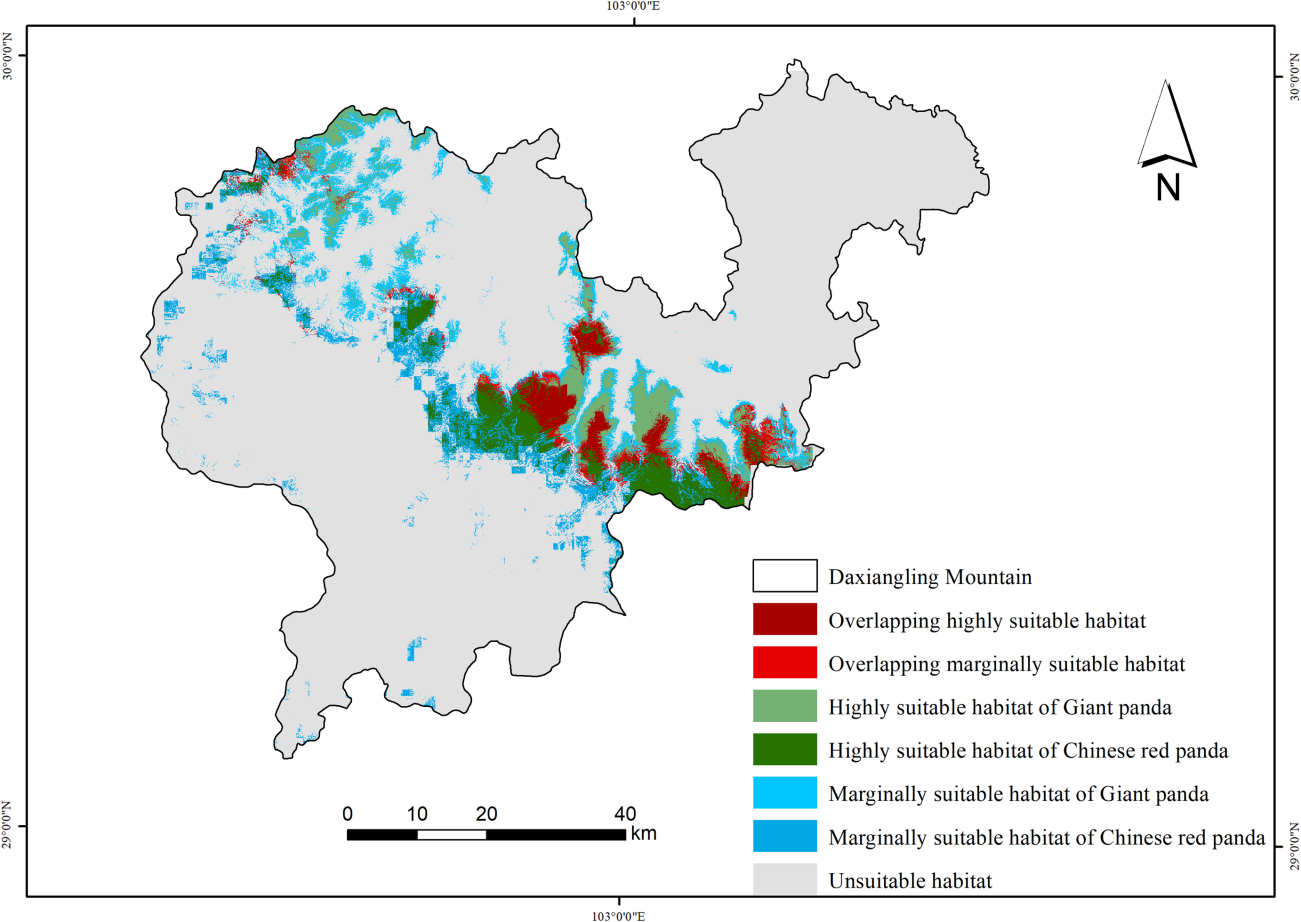
Habitat overlap of Giant pandas and Chinese red pandas in Daxiangling mountain.

The total overlapping area of giant panda and Chinese red panda habitats was 342.23 km^2^, of which the overlapping area of highly suitable habitats was 98.91 km^2^, accounting for 47.69% of the total area of giant panda habitats and 46.88% of the total area of Chinese red panda habitats; The overlapping area of highly suitable habitats accounted for 13.78% of the area of suitable habitats for giant pandas and 13.55% of the area of suitable habitats for Chinese red pandas (Figure 6). From the point of view of spatial niche overlap index, the habitat overlap index is 0.472, and the high suitable habitat overlap index was 0.348. The high suitable habitat overlap index was smaller than the suitable habitat overlap index. The overlap degree of highly suitable habitats of giant pandas and Chinese red pandas was less than that of suitable habitats, which indicated that the two achieve spatial niches were separated for avoiding highly suitable habitat overlap to achieve stable coexistence.

### 3.2 Temporal niche

#### 3.2.1 Activity rhythm analysis

**FIGURE 7:**
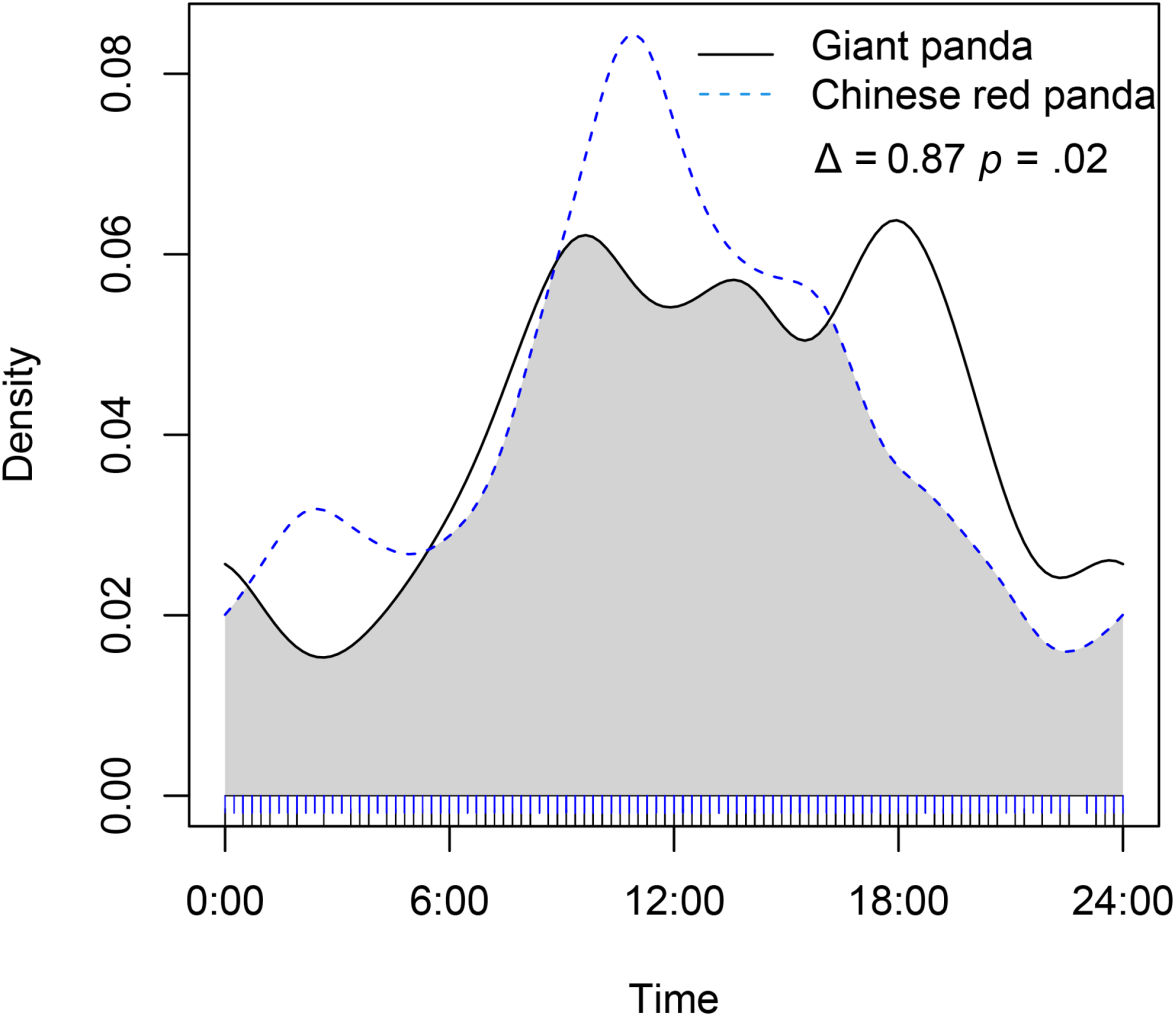
The daily activity rhythms of giant pandas and Chinese red pandas.

Both giant pandas and Chinese red pandas showed obvious diurnal behavior and maintain high activity intensity in the daytime, but their activity peaks were quite different (Figure 7). The two activity peaks of giant pandas were more obvious, respectively, around 8:00 and 18:00, and the activity peak of Chinese red pandas was around 11:00. In terms of time niche overlap, the overlap index of the daily activity rhythm of giant pandas and Chinese red pandas was 0.87, and Wald test results showed that there was a significant difference between them (*p* < .05).

#### 3.2.2 Activity rhythm interaction

**FIGURE 8:**
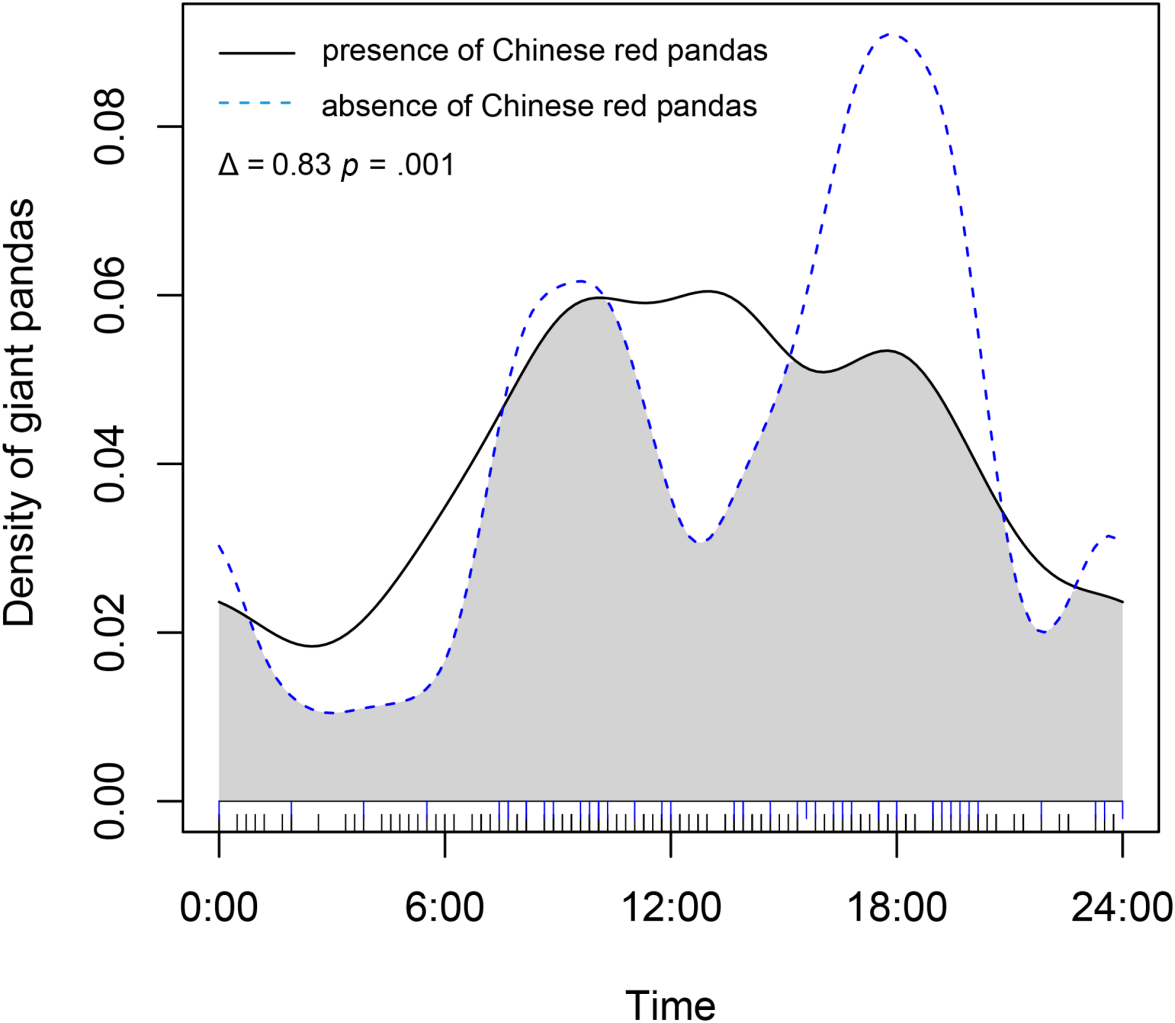
The activity rhythm of the giant pandas in the presence or absence of Chinese red pandas.

**FIGURE 9:**
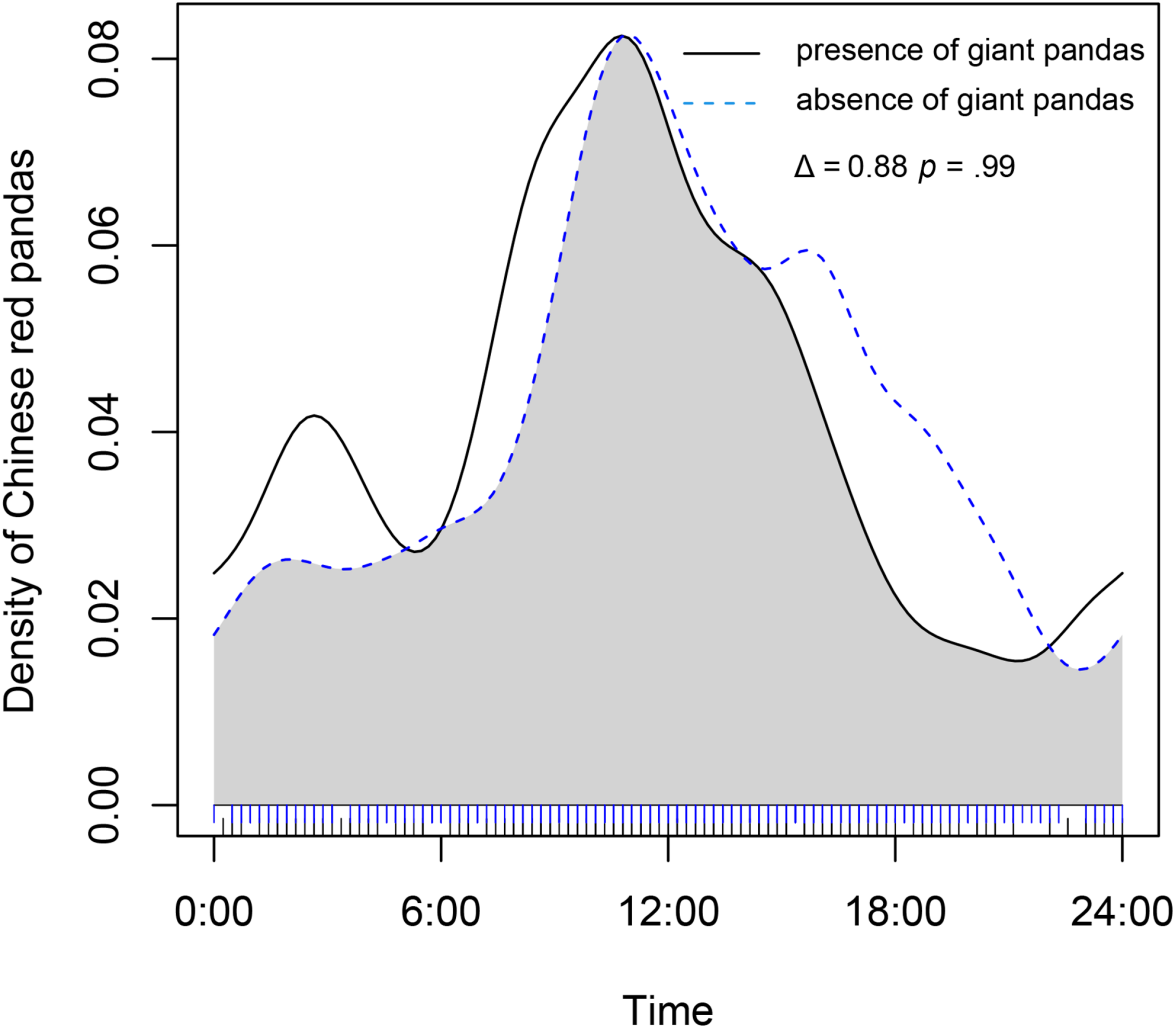
The activity rhythm of the Chinese red pandas in the presence or absence of giant pandas.

The daily activity rhythm overlaps the index of giant pandas in the presence of Chinese red pandas and in the absence of Chinese red pandas was 0.83 (Figure 8). Wald test results showed that the presence of Chinese red pandas may have a significant impact on the daily activity rhythm of giant pandas (*p* = .001). In the presence of Chinese red pandas, the activity intensity of giant pandas increased from 0:00 to 14:00, while that of late peak (16:00–20:00) decreased significantly. In the presence of giant panda and the absence of giant pandas, the overlap index of the daily activity rhythm of Chinese red pandas was 0.88 (Figure 9). The Wald test results showed that the presence or absence of giant pandas did not have a significant impact on the daily activity rhythm of Chinese red pandas (*p* = .99). It indicated that the presence or absence of giant pandas will not significantly affect the daily activity rhythm of Chinese red pandas. In the presence of giant pandas, Chinese red pandas increased their diurnal activity intensity (23:00–11:00) but decreased their activity intensity (11:00–23:00).

## 4 DISCUSSION

Spatial niche is the basis for understanding the regional coexistence and interaction of sympatric species. Only when species coexist in a certain space can they potentially interact in other niche dimensions such as time and nutrition (Farris et al., 2020). Habitat utilization of giant pandas and Chinese red pandas distributed in the same region may be affected by many factors, such as resource acquisition, predation risk, interspecific competition, human interference, etc. (Bai et al., 2018; Dong et al., 2021). In the Daxiangling Mountains, the suitable habitats for giant pandas and Chinese red pandas are highly overlapped, with the overlapping area of 47.69% and 46.88% of their total habitat area, respectively. There are still some nonoverlapping habitats, where the activity rhythm of species might be similar to this trend of the absence of sympatric species in the overlapping habitats. However, the difference in space use can effectively promote the coexistence of species, and the selection and utilization of specific resources is an ecological behavior of wildlife to adapt to the environment. In this study, the suitable habitat and high degree of overlap of giant pandas and Chinese red pandas indicated that the two achieve spatial niches were separated for avoiding a high degree of overlap of highly suitable habitats to achieve stable coexistence.

Studies have shown that giant pandas and Chinese red pandas are selective to the gradient, vegetation type, aspect, elevation, water source, human disturbance, and other factors of their habitats, and their distribution will also be affected by these factors (Dong et al., 2021; Yang et al., 2017). There are studies that have shown giant pandas preferred to select the habitat with the gentle slope facing to the south with dense forest canopy. On the other hand, Chinese red pandas showed a strong habitat preference for deep slope facing to the south with dense canopy (Wei et al., 1999). In addition, they all preferred to stay away from human disturbances such as roads, which is consistent with our results. Our results also showed that they avoid high‐intensity competition by living in different environments in the Daxiangling Mountains. For example, giant pandas can withstand higher temperature seasonal changes (Bio4); Giant pandas prefer coniferous forests, broad‐leaved forests, and shrubs, while Chinese red pandas prefer coniferous forests and mixed coniferous and broad‐leaved forests. These differences may also be important for maintaining their mutual adaptation and long‐term coexistence. Although habitat separation can avoid interspecific competition to the greatest extent, the animals that form habitat separation will necessarily abandon part of the better‐quality habitat, so the animals will make a trade‐off between using the better habitat and mitigating the pressure of interspecific competition (Broekhuis et al., 2013).

When the spatial niches of sympatric species overlap, they will separate into other niche dimensions, so as to reduce the overlap of multidimensional niches and maintain the coexistence of species (Bagchi et al., 2003). In this study, the spatial niche and temporal niche of giant panda and Chinese red panda were differentiated to different degrees, and they had made some trade‐offs in the selection of spatial and temporal niches. When the two appeared in the same region, the interspecific competition was inevitable. The competition between giant pandas and Chinese red pandas belongs to the resource utilization competition (Kronfeld‐Schor & Dayan, 2003). When the two appear in the same area, they both improve their competitiveness on the resource by changing the activity rhythm. Both species choose to increase activities from morning to afternoon and reduce activities from evening to morning, indicating that they are in competition for high‐quality resources. In addition, other sympatric species, such as Asiatic black bears (*Ursus thibetanus*), Tibetan macaque (*Macaca thibetana*), and other animals, may also participate in this competition, which has an impact on the spatiotemporal ecological niche of giant pandas and Chinese red pandas (Bista et al., 2018). However, the food resources in fixed areas are limited. When there is competition, animals will inevitably adjust to have a higher activity intensity to obtain food resources sufficient to support life activities. In order to maintain the balance of energy expenditure, the activity intensity of competing for high‐quality food resources is increased, and the activity intensity of the rest of the time is correspondingly reduced.

As important components of the forest ecosystem, giant pandas and Chinese red pandas are the flagship species for biodiversity conservation in the mountain forests of southwest China, which are of great significance for maintaining regional biodiversity and ecosystem stability (Li & Pimm, 2015). Our research showed that there is still a large area of suitable habitat for Chinese red pandas outside the protected area, and it does not overlap with the habitat of giant pandas. In addition, in the Daxiangling Mountains, the habitats of giant pandas and Chinese red pandas are obviously fragmented. A few patches are highly isolated, and the suitable habitats of giant pandas are more fragmented and less connected. It is necessary to strengthen the restoration of fragmented habitats and connectivity management for the protection of rare animals such as giant pandas and Chinese red pandas, which will help to increase the suitable habitats for giant pandas and Chinese red pandas and promote the spread and exchange of individuals (Haddad et al., 2015; Smith & Rulifson, 2015; Wang, Winkler, et al., 2021). We suggest to formulate corresponding protection measures according to the habitat distribution of each species and the preference of environmental factors, and then promote the protection of multiple species, so as to achieve the comprehensive protection and management of more species distributed in the same region, and optimize the umbrella effect on other species while protecting giant pandas.

## 5 CONCLUSION

In the Daxiangling Mountains, the total area of suitable habitats for giant pandas and Chinese red pandas is 717.61 and 730.00 km^2^, respectively, accounting for 17.78% and 18.25%, respectively, of the study area. The total overlapping area of suitable habitats for giant pandas and Chinese red pandas is 342.23 km^2^, of which the overlapping area of highly suitable habitats is 98.91 km^2^. The overlapping index of suitable habitats is 0.472, and the overlapping index of highly suitable habitats is 0.348, which indicates that the two achieve spatial niches are separated to achieve stable coexistence. The top five environmental factors contributing to the model of giant panda and Chinese red panda are precipitation seasonality, temperature seasonality, distance to the road, and elevation and vegetation type. The overlapping index of the daily activity rhythm of giant panda and Chinese red panda is 0.87, which is significantly different (*p* < .05). The existence of Chinese red panda will significantly affect the daily activity rhythm of giant panda (*p* < .001).

## AUTHOR CONTRIBUTIONS

**Bin Feng:** Data curation (equal); formal analysis (equal); writing – original draft (equal). **Wenke Bai:** Data curation (equal); formal analysis (equal); resources (equal); writing – original draft (equal). **Xueyang Fan:** Writing – review and editing (equal). **Mingxia Fu:** Data curation (equal). **Xinqiang Song:** Data curation (equal). **Jingyi Liu:** Data curation (equal). **Weirui Qin:** Data curation (equal). **Jindong Zhang:** Writing – review and editing (equal). **Dunwu Qi:** Writing – review and editing (equal). **Rong Hou:** Resources (equal); supervision (equal); writing – review and editing (equal).

## CONFLICT OF INTEREST STATEMENT

The authors declare no conflict of interest.

## Data Availability

Related data are openly available in Dryad at https://doi.org/10.5061/dryad.3bk3j9kpd.
